# Hfm1 participates in Golgi-associated spindle assembly and division in mouse oocyte meiosis

**DOI:** 10.1038/s41419-020-2697-4

**Published:** 2020-06-30

**Authors:** Huiyuan Wang, Chenyi Zhong, Rui Yang, Yaoxue Yin, Rongrong Tan, Li Gao, Chao Gao, Yugui Cui, Danhua Pu, Jie Wu

**Affiliations:** State Key Laboratory of Reproductive Medicine, Department of Obstetrics and Gynecology, The First Affiliated Hospital of Nanjing Medical University, Jiangsu Province Hospital, Jiangsu Women and Children Health Hospital, Nanjing, China

**Keywords:** Oogenesis, Endocrine reproductive disorders, Infertility, Disease genetics

## Abstract

HFM1 (helicase for meiosis 1) is widely recognized as an ATP-dependent DNA helicase and is expressed mainly in germ-line cells. HFM1 is a candidate gene of premature ovarian failure (POF), hence it is also known as POF9. However, the roles of HFM1 in mammalian oocytes remain uncertain. To investigate the functions of HFM1, we established a conditional knockout (cKO) mouse model. Specific knockout of Hfm1 in mouse oocytes from the primordial follicle stage resulted in depletion of ovarian follicular reserve and subfertility of mice. In particular, abnormal spindle, misaligned chromosomes, loss of cortical actin cap, and failing polar body extrusion were readily observed in Hfm1-cKO oocytes. Further studies indicated that in addition to its cytoplasmic distribution, Hfm1 accumulated at the spindle poles, colocalized with the Golgi marker protein, GM130. Generally, GM130 signals overlapped with p-Mapk at the two spindle poles to regulate meiotic spindle assembly and asymmetric division. In this research, centrosome associated proteins, such as GM130 and p-Mapk, detached from the spindle poles in Hfm1-cKO oocytes. In conclusion, our data suggest that Hfm1 participates in Golgi-associated spindle assembly and division in mouse oocyte meiosis. These findings provide clues for pathogenesis of POF.

## Introduction

HFM1 (helicase for meiosis 1), also known as MER3, POF9, Si-11, SEC63D1, and Si-11-6, is regarded to be an ATP-dependent DNA helicase and is expressed mainly in germ-line cells^1^. POF9 is derived from premature ovarian failure, which is now commonly named as primary ovarian insufficiency (POI) or premature ovarian insufficiency (POI). POI is characterized by ovarian impairment/failure before the age of 40 years, which is a major cause of female infertility^2^. The etiology of POI is complicated that although substantial evidence suggests a genetic basis for POI, the majority of cases remain unexplained^3^. In 2014, exome sequencing of samples obtained from two Chinese sisters with POI and their parents identified a shared compound heterozygous mutation in HFM1. A verification study was then performed in a small sample size of sporadic POI and controls, in which another HFM1 compound heterozygous mutation was identified in one case^4^. Our previous research also proved that the mutation rate of HFM1 in sporadic POI patients was significantly higher than that in controls and six different variants of HFM1 gene were identified in POI group^5^. A novel heterozygous splice-altering mutation have also been reported recently^6^, which further confirmed that the mutation of the HFM1 gene might be associated with POI in Chinese population.

Previous Hfm1 knockout mice model illustrated that Hfm1 is required to form normal numbers of crossovers and to complete synapsis in spermatogenesis process^7^. Correspondingly, direct sequencing of the coding regions in HFM1 gene proved that HFM1 gene variants may associate with idiopathic oligo/azoospermia in Chinese men^8^. Moreover, female mice showed a significant reduction in ovary size and a decreased number of follicles^7^. However, it is not clear how Hfm1 participates in oogenesis process.

Oocyte maturation is crucial to female reproductive biology, and it is a complex process that includes both nuclear and cytoplasmic maturation^9^. Nuclear maturation mainly includes resumption of meiosis I, polar body extrusion, and maintaining metaphase of meiosis II (MII) stage. In addition, cytoplasmic maturation involves organelles reorganization, the mRNA, proteins storage, and transcriptional regulation^10,11^. The synchrony between nuclear and cytoplasmic maturation regulates oocyte asymmetric division by spindle assembly and migration, which is a unique hallmark of meiosis^12^. Unlike mitotic cells, mammalian oocytes lack typical centrosomes, but contain multiple microtubule organizing centers (MTOCs), a complex of centrosome proteins that form the spindle poles and organize the microtubule network^13^. Some Golgi apparatus proteins have unexpected roles in MTOC formation^14^. The most prominent one is GM130, a cis-Golgi protein, which is particularly enriched in the spindle poles at both MI and MII stages, regulating spindle organization, migration, and asymmetric division during oocyte maturation^15,16^.

To date, HFM1 may be a compelling candidate gene for POI^17^, but its explicit role in oocyte meiosis remains unclear. In this study, we aimed to investigate the detailed functions of Hfm1 during mouse oocyte maturation by utilizing an oocyte specific Hfm1 knockout mouse model and hoped to provide novel clues for the pathogenesis of POI.

## Results

### Oocyte-specific knockout of Hfm1 in mice

**Fig. 1:**
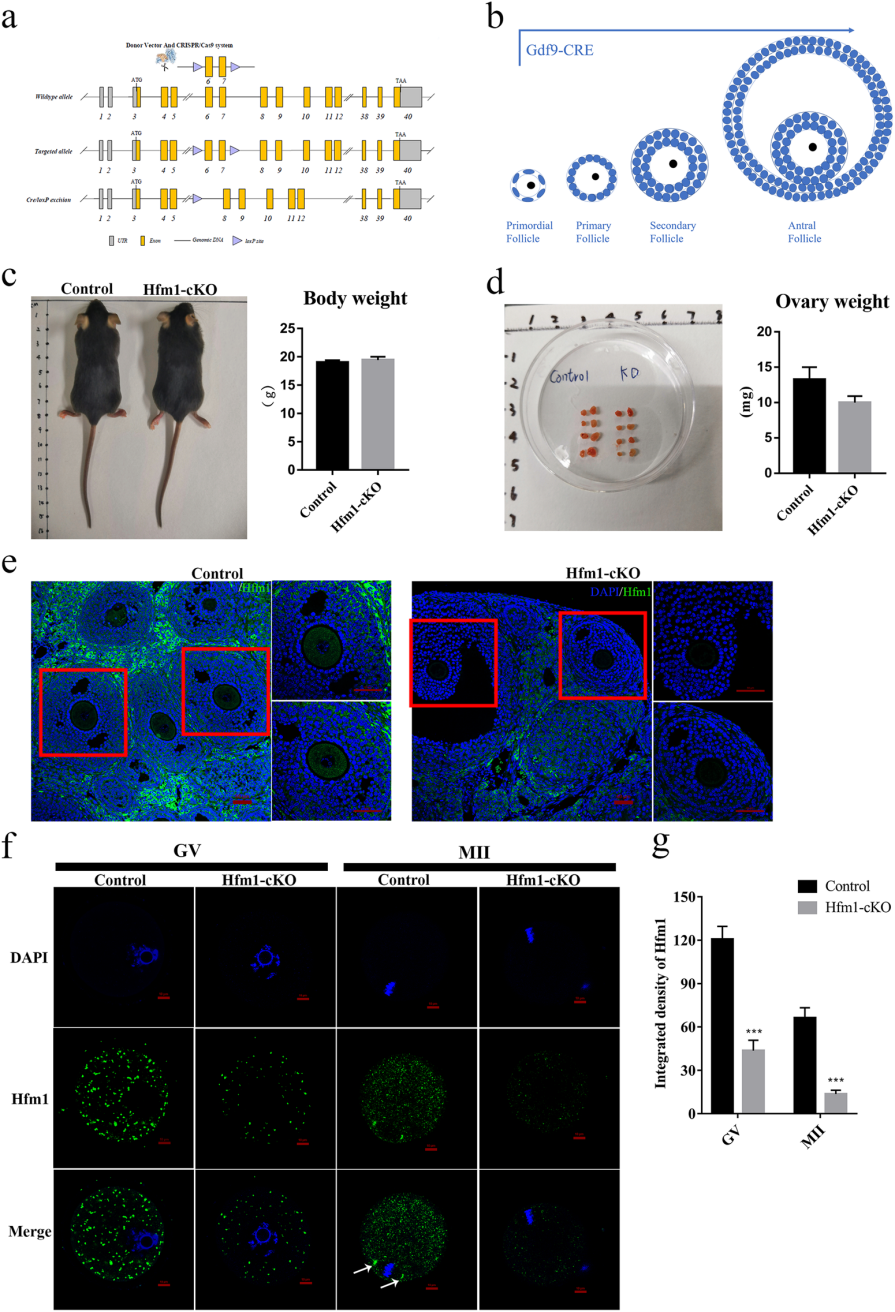
Oocyte-specific Knockout of Hfm1 in mice. **a** Engineered a conditional floxed allele for Hfm1 and a Cre-mediated recombination to delete exons 6 and 7. **b** Schematic illustration of Gdf9-Cre-mediated Hfm1 knockout in oocytes from primordial follicle stage **c** Photograph of whole bodies and measured the body weight of Control and Hfm1-cKO mice (2 months old) (*n* = 4). **d** Photograph of a pair of ovaries and measured the ovaries weight of Control and Hfm1-cKO mice (2-month-old) (*n* = 4). **e** Immunofluorescence staining of Hfm1 in ovaries of Control and Hfm1-cKO mice (2-month-old). **f** Immunofluorescence showed expression levels and subcellular localization of Hfm1 (green) in GV and MII oocytes of Control and Hfm1-cKO mice (2-month-old). DAPI are co-stained to visualize DNA (blue). White arrows point to spindle pole in oocytes. GV, Germinal Vesicle; MII, metaphase of meiosis II. **g** Quantification of the Hfm1 staining (*n* = 10). ***P < 0.01 by two-tailed Student’s *t* tests. Data represent the mean ± SEM.

To assess the role of Hfm1 during oocyte development, mice carrying Gdf9-Cre transgenes and a floxed allele of Hfm1 were designed to delete exons 6 and 7 (Fig. 1a), which express the Cre recombinase specifically in oocytes from primordial follicle stage^18^ (Fig. 1b). The Hfm1^fl/fl^, Gdf9-Cre female mice were referred to as Hfm1-cKO and Hfm1^fl/fl^ female mice were referred to as Control. The body weight (Fig. 1c) and ovary weight (Fig. 1d) of young adult (2 months old) Hfm1-cKO mice were comparable to control group. To verify that Hfm1 was deleted in Hfm1-cKO oocytes, histology immunofluorescence was performed and showed that Hfm1 was nearly undetectable in oocytes of Hfm1-cKO ovaries (Fig. 1e). Furthermore, we isolated fully grown oocytes from mice and performed single-oocyte immunofluorescence (Fig. 1f). The expression level of Hfm1 in Hfm1-cKO oocytes showed an ~70% reduction compared with controls (Fig. 1g), and Hfm1 protein was enriched in the spindle poles in MII oocytes (Fig. 1f, white arrow).

### Compromised fertility in Hfm1-cKO mice

**Fig. 2:**
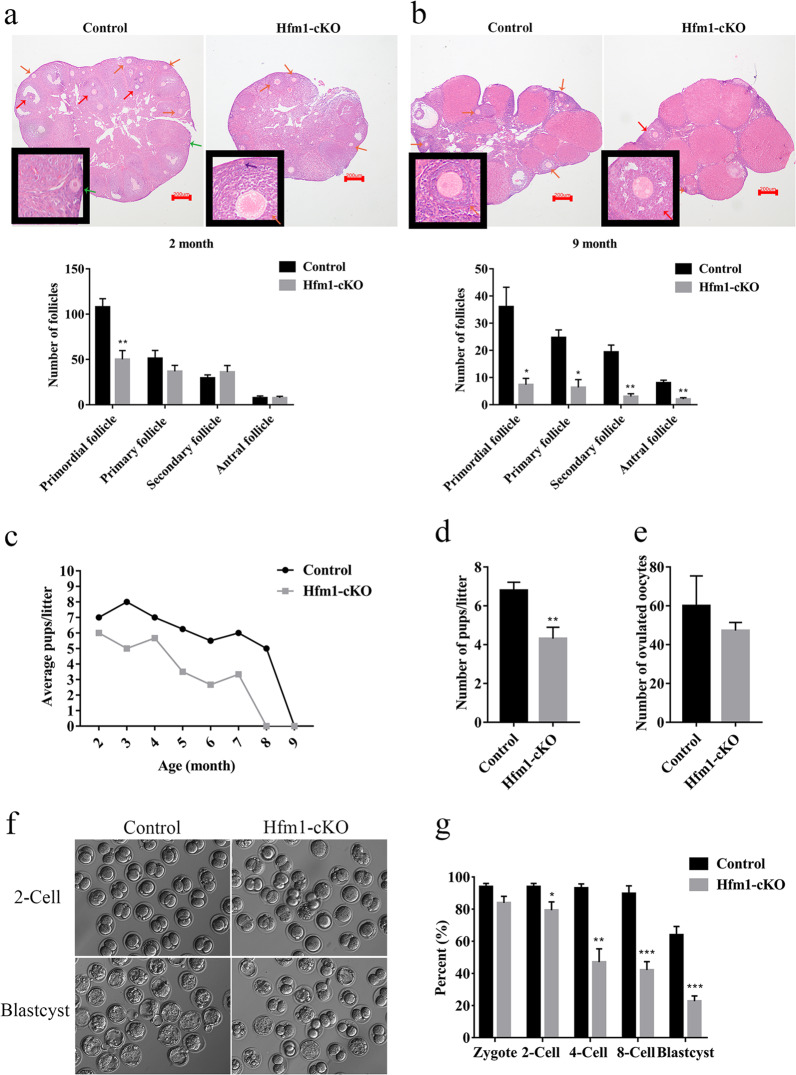
Compromised fertility in Hfm1-cKO mice. **a** H&E stained and follicle quantification in ovaries from adult Control and Hfm1-cKO mice (2-month-old) (*n* = 5). **b** H&E stained and follicle quantification in ovaries from older Control and Hfm1-cKO mice (9-month-old) (*n* = 4). The primordial (purple arrow), primary (green arrow), secondary (orange arrow) and antral follicles (red arrow) were annotated, and count data were presented per ovary. **c** Average pups numbers of each litter per month in Control and Hfm1-cKO mice (2-month-old) (*n* = 4). **d** The quantification analysis of pups per litter by the bar graph in a standard 7 months breeding trial. **e** Number of oocytes ovulated by prepubertal Control and Hfm1-cKO females (3-week-old) (*n* = 4). **f** Embryo developmental potential of oocytes before implantation from Control and Hfm1-cKO females (3-week-old) assessed by IVF. **g** The rate of zygote, 2-Cell, 4-Cell, 8-Cell, and blastocyst formation normalized to the number of MII oocytes ovulated by Control and Hfm1-cKO females (3-week-old) during IVF (*n* = 4). *P < 0.05. **P < 0.01. ***P < 0.001. Significance was determined by two-tailed Student’s t tests. Data represent the mean ± SEM.

To confirm whether Hfm1-cKO influenced reproductive function, follicle counts of all stages, including primordial, primary, secondary, and antral follicles, were recorded according to acknowledged morphological criteria^19^. In young adult mice (2-month-old), Hfm1-cKO ovaries showed a significant decrease in primordial follicles (*P* < 0.01), but no difference in growing follicles (primary, secondary and antral follicles) compared with control group (Fig. 2a). However, in older female mice (9-month-old), all stages follicles decreased significantly in Hfm1-cKO females compared with controls (Fig. 2b).

Despite no overt decrease was observed in growing follicles of Hfm1-cKO young adult mice, fertility competence was further evaluated to validated whether there were any alterations in oogenesis. Fertility testing revealed that, Hfm1-cKO female mice were premature infertile (Fig. 2c) and produced about four pups per litter on average, which was significantly lower than about seven pups per litter in control group during 7 months breeding (Fig. 2d). However, prepubertal Hfm1-cKO mice (3-week-old) were over-ovulated an average of 47 oocytes, which had no difference with an average of 60 oocytes in control group (Fig. 2e). Furthermore, in vitro fertilization (IVF) testing were performed with normal sperms. The results suggested that about 83% of the Hfm1-cKO mice ovulated oocytes underwent successful fertilization, but 30% or less of the fertilized Hfm1-cKO eggs developed to blastocysts in culture, which were significantly lower than that in control group (94% fertilization and 65% developed to blastocysts) (Fig. 2f, g). In sum, reproductive potential was severely compromised in Hfm1-cKO female mice.

### Abnormal spindle in Hfm1 knockout oocytes

**Fig. 3:**
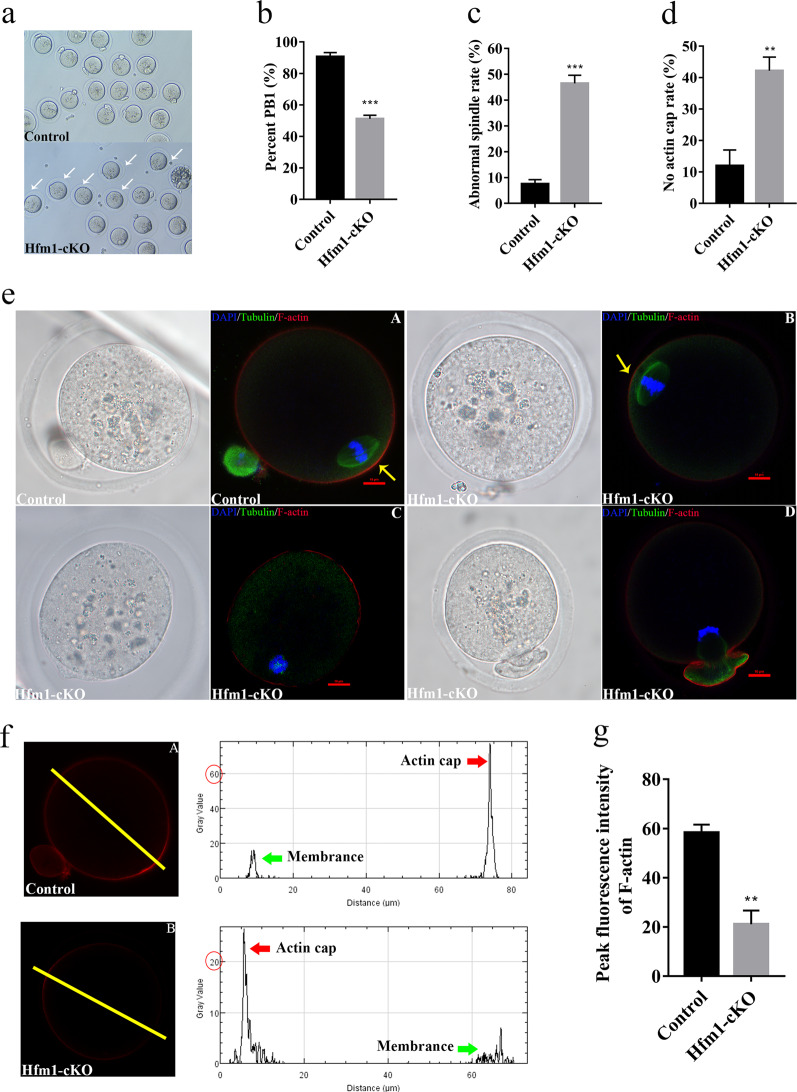
Abnormal spindle in Hfm1 knockout oocytes. **a** The micrographs of oocytes from Control and Hfm1-cKO females (3-week-old) after IVM. White arrows point to no first polar body (PB1) extrusion oocytes. **b** The PB1 extrusion rate of oocytes from Control and Hfm1-cKO females (3-week-old) by IVM (*n* = 4). **c** Percentage of in vivo matured MII oocytes with abnormal spindle from Control and Hfm1-cKO females (3-week-old) (*n* = 3). **d** Percentage of in vivo matured MII oocytes with no actin cap from Control and Hfm1-cKO females (3-week-old) (*n* = 3). **e** Representative spindles from MII oocytes ovulated by Control and Hfm1-cKO females (3-week-old) in vivo. (A) Normal MII stage. (B) abnormal MII with a spindle and a decreased actin cap fluorescence intensity. (C) Abnormal MII with no spindle and no actin cap. (D) Abnormal MII with incomplete division spindle and no actin cap. Microtubules, Chromosomes, and F-actin are stained green, blue, and red, respectively. Yellow arrows pointed to actin cap. **f** Profiles of phalloidin fluorescence intensity along the yellow line in oocytes (A) and (B). The yellow line perpendicular to the length axis of the spindle **g** Quantitative analysis of peak actin immunofluorescence intensities in profiles of Control and Hfm1-cKO oocytes with actin cap (*n* = 10). *P < 0.05. **P < 0.01. ***P < 0.001. Significance was determined by two-tailed Student’s *t* tests. Data represent the mean ± SEM.

To investigate the potential mechanism of subfertility in Hfm1-cKO mouse, oocyte meiotic progression was examined because meiotic errors reduced oocyte quality and oocyte competency is the key to embryo potential^20^. Therefore, we collected fully grown oocytes from superovulated female mice of each genotype and performed in vitro maturation (IVM) (Fig. 3a). Only about 50% Hfm1 knockout oocytes produced the first polar body, which was significant lower than ~90% in control group (Fig. 3b). In vivo mature MII oocytes were assessed for spindle morphology by immunofluorescence, and about 45% mutant oocytes demonstrated abnormal spindles while only 7% in control group (Fig. 3c). The major defect in Hfm1-cKO oocytes was no spindle formation (Fig. 3e-C), and other abnormities included incomplete spindle division (Fig. 3e-D), spindles with no poles and deformed spindles with astral microtubules. In addition, spindle migration is an actin-related process^21^. Actin cap formation was disrupted in about 40% Hfm1 knockout oocytes, which was much higher than that in control oocytes (Fig. 3d), and actin filament signals at the cortex decreased visibly in Hfm1-cKO oocytes (Fig. 3f, g).

### Hfm1 functions in Golgi-associated spindle assembly and division

**Fig. 4:**
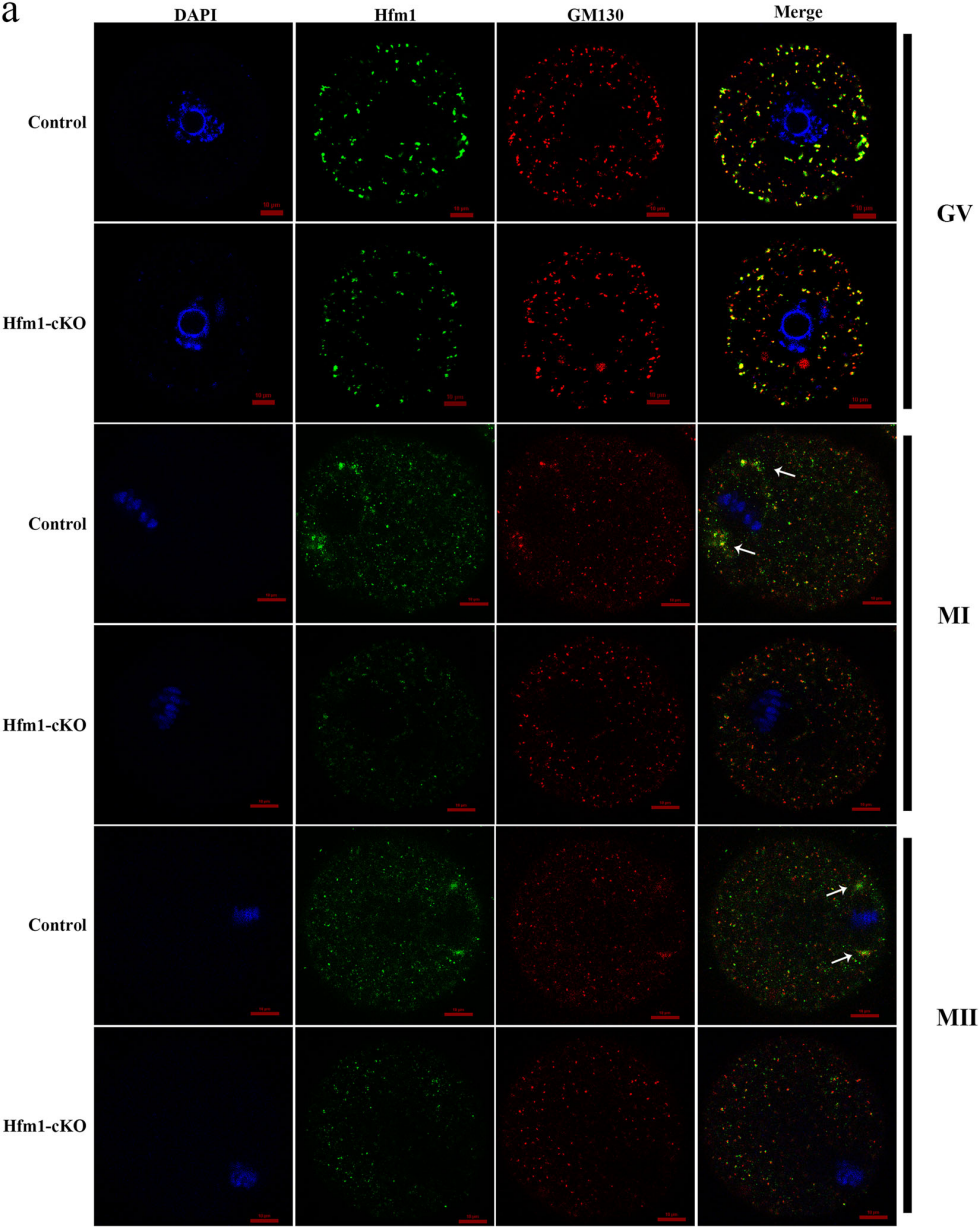
Hfm1 functions in Golgi-associated spindle assembly and division. **a** Double immunofluorescent staining of Hfm1 (green) and GM130 (red) in GV, MI and MII stage. DAPI are co-stained to visualize DNA (blue). White arrows point to spindle pole in oocytes. GV germinal vesicle, MI metaphase of meiosis I, MII metaphase of meiosis II.

As shown in Fig. 1f, in germinal vesicle (GV) stage control oocytes, Hfm1 was dispersed throughout the cytoplasm and seemingly concentrated in the interior than at the cortex. In MII stage control oocytes, Hfm1 was localized at the spindle poles in a crescent shape (white arrow). The apparent redistribution of Hfm1 was similar with the reorganization of Golgi apparatus in oocyte cytoplasmic maturation. To confirm whether Hfm1 was co-localized with the Golgi apparatus, double immunofluorescent staining was preformed including Hfm1 and known Golgi marker-GM130. Hfm1 followed the same localization pattern as that of GM130 and their signals overlapped at GV, MI, and MII stage (Fig.4a). As is known, GM130 accumulated at the spindle poles in MI and MII stages^15,22^, but the localization of GM130 was altered and dispersed into the cytoplasm in Hfm1-cKO oocytes (Fig.4a, white arrow).

**Fig. 5:**
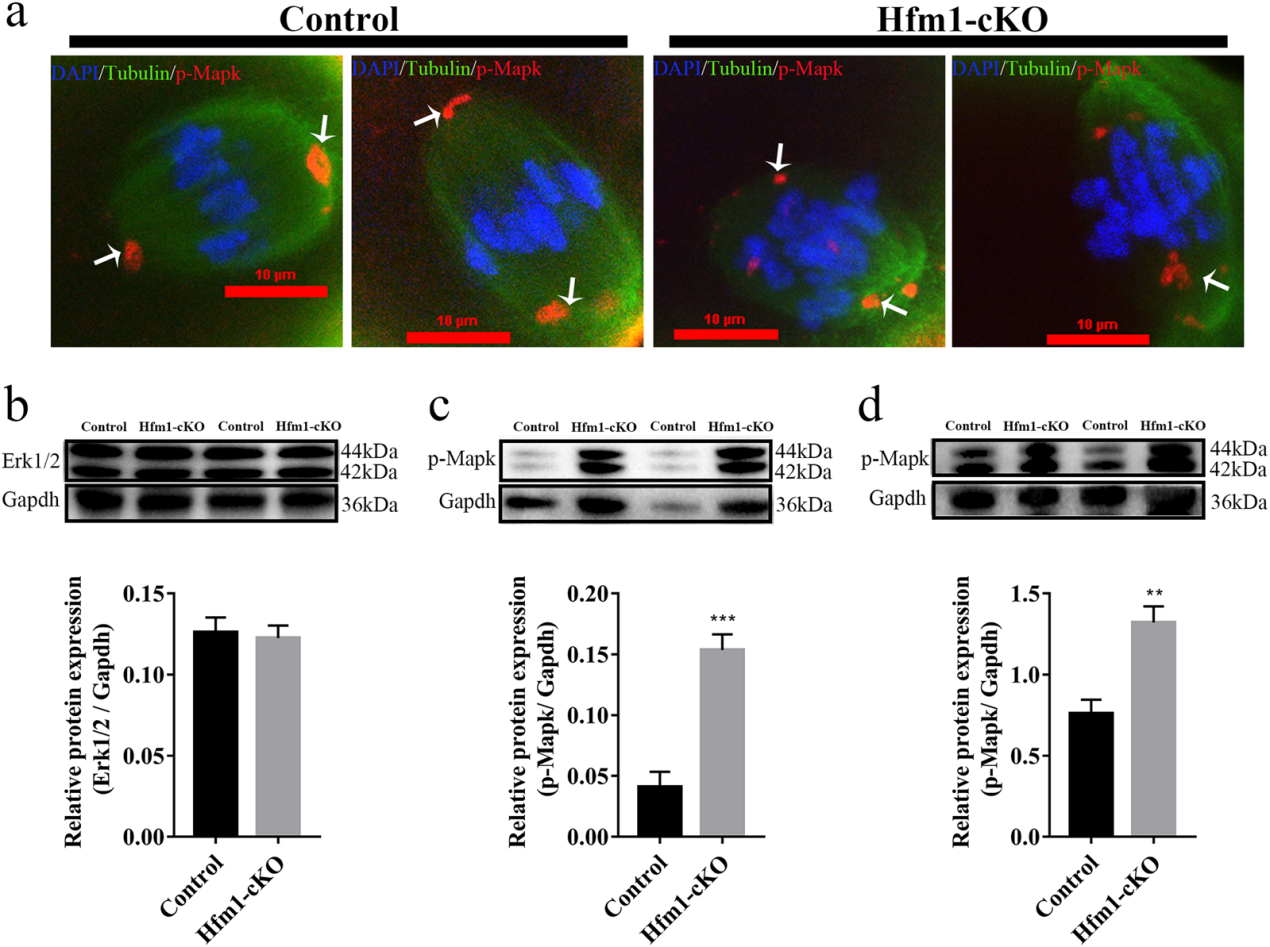
Disrupted MAPK pathway in Hfm1 knockout oocytes. **a** Denuded oocytes cultured for 8 h (MI) and stained with p-Mapk (red), β-Tubulin (green), and DAPI (blue). White arrows point to p-Mapk signals in oocytes. **b** Western blot analysis of Erk1/2 expression in Control and Hfm1-cKO ovaries (*n* = 5). **c** Western blot analysis of p-Mapk expression in Control and Hfm1-cKO ovaries (*n* = 5). **d** Western blot analysis of p-Mapk in Control and Hfm1-cKO oocytes at MI stage (*n* = 4). Protein lysates from 100 oocytes were loaded in each lane. **P < 0.01. ***P < 0.001. Significance was determined by two-tailed Student’s *t* tests. Data represent the mean ± SEM.

GM130 might cooperate with the MAPK pathway to participate in spindle organization, migration, and asymmetric division^15,23^. To further determine the involvement of Hfm1 in spindle formation, the localization of phospho-p44/42 mitogen activated protein kinase (p-Mapk) was measured, which was proved enriched in spindle poles and crucial for proper spindle formation during oocyte meiosis^23,24^. After GV stage oocytes cultured for 8 h, p-Mapk was localized at spindle poles in control MI oocytes, whereas p-Mapk detached from spindle poles and was detected on the spindle fibers or dispersed to the cytoplasm in Hfm1-cKO MI oocytes (Fig. 5a, white arrow). Moreover, misaligned chromosomes were also observed in Hfm1-cKO MI oocytes (Fig. 5a). Western Blot analysis showed that the expression levels of Erk1/2 in Hfm1-cKO ovaries were comparable to that of control ovaries (Fig. 5b), while the expression levels of p-Mapk were significantly higher in Hfm1-cKO ovaries (Fig. 5c). Moreover, the expressions of p-Mapk in Hfm1 knockout MI oocytes were upregulated as well compared with controls (Fig. 5d).

## Discussion

The current study was designed to explore the role of Hfm1 involving in mouse oocyte maturation. In this research, we found that Hfm1 special deletion from primordial follicle stage oocytes led to accelerated exhaustion of the ovarian follicular reserve in mice, which may related to premature reproductive aging (Fig. 2a, b). The irregular follicle development compromised fertility (Fig. 2c, d) and affected oocyte quality in female mice (Fig. 2f, g).

As was observed, the preimplantation embryo developmental capacity gradually decreased in Hfm1-cKO group, along with increasing number of cell division times (Fig. 2f, g), which implied a clue to spindle apparatus. Oocytes with absent or abnormal spindles, if fertilized, have a lower potential to form normal embryos^25^. In clinical settings, gene mutations may lead to meiotic defects and then impair early embryonic development^26^. During oocyte meiotic maturation, Hfm1 was partly concentrated at oocyte spindle poles (Fig. 1f, white arrow). Even though prepubertal Hfm1-cKO mice ovulated a similar number of oocytes in comparison to control group (Fig. 2e), the first polar body extrusion rate decreased visibly in IVM testing (Fig. 3a, b). To exclude the interference of in vitro culture conditions, spindle morphology was explored using in vivo mature MII oocytes. Results of immunofluorescence showed that about 45% oocytes ovulated by Hfm1-cKO mice neither completed the first meiosis, nor formed MII spindles normally (Fig. 3c, e). Strikingly, actin cap, an actin enriched zone in the cortex during MII, was disrupted in Hfm1-cKO oocyte (Fig. 3e, yellow arrow), together with actin signals at the cortex was significantly reduced in Hfm1 deletion oocytes compared with controls (Fig. 3f, g). Recent works have revealed that oocyte spindles require not only microtubules but also actin to promote asymmetric division^27^. Actin dynamic networks are indispensable to spindle formation and migration^28,29^. Collectively, all of these results indicated that the failure of oocyte maturation might be due to aberrant spindle assembly and division.

Over the past decade, oocyte nuclear maturation has been well studied, while cytoplasmic maturation is still controversial. The proper temporal and spatial dynamics of organelles and the cytoskeleton must occur for the oocyte to ensure oocyte competency, which is a key to fertilization and subsequent embryo development^20^. The Golgi apparatus is not only a chief in protein modified and transferred, but also an anchor to microtubule minus ends, participating in cell polarization, migration, and division^30,31^. In GV mouse oocytes, the Golgi apparatus spreads among the cytoplasm in the form of a continuous membranous system and slightly more concentrated in the interior than at the cortex. Then, the Golgi apparatus is fragmented and dispersed in MI oocytes, and this debris state is maintained following extrusion of the first polar body in MII oocytes^32^. Notably, GM130, a marker of the Golgi apparatus, localizes at the spindle poles at both MI and MII stages (Fig. 4a, white arrow) and plays a role in spindle organization, migration, and asymmetric division during mouse oocyte maturation^15^. Hfm1 had a similar localization pattern with GM130 (Fig. 4a) in this study, which may hint at a functional mechanism. In addition, a recent study has demonstrated that vesicle-based actin network is essential for asymmetric spindle positioning and polar body extrusion in mouse oocytes^33^, while the vesicles are derived from Golgi complexes during oocyte growth. Actin and associated proteins play a significant role in Golgi structure and function^34^. Hence, we hypothesized that Hfm1 might participate in Golgi-associated spindle assembly and division in mouse oocyte meiosis.

The spindle apparatus is responsible for separating homologous chromosomes during meiosis I and sister chromatids during meiosis II to produce haploid oocytes^35^. Despite the absence of common centrosomes, oocytes express many centrosomal proteins. Some of these proteins, such as GM130, p-Mapk, γ-Tubulin, and Plk1, have been mapped to the centriolar MTOCs, which functionally replace centrosomes to form the spindle poles and organize the microtubule network in mouse oocytes^36^. Commonly, the two sister kinetochores of each chromosome are paired and face toward the same spindle pole during meiosis I, but Hfm1-cKO oocyte showed misaligned chromosomes (Fig. 5a). To further confirm the involvement of Hfm1 in spindle assembly and division, the situation of p-Mapk was examined, which has been demonstrated enriched in spindle poles and essential for proper spindle formation during oocyte meiosis^24,37^. As shown in Fig. 5a–d, Hfm1 knockout resulted in the disrupted localization and the increased expression of p-MAPK in MI oocytes. Moreover, the Hfm1-cKO ovaries showed MAPK pathway excessive activation, which provided us a hint that Hfm1 may participate in the MAPK signaling pathway to regulate the spindle migration and asymmetric division. Although several studies have reported that MAPK signaling pathway was indispensable to the maturation of the mouse oocyte^38^ and can regulate the Golgi-associated spindle migration and asymmetric division in oocyte meiosis^15,23,28^, the present study was the first time to point out that Hfm1 may involve in this process. However, the detailed mechanism of Hfm1 in this ambiguous network must be further explored in the future.

Up to now, the complete functions and mechanisms of Hfm1 in oogenesis are still unclear. Here, we revealed the role of Hfm1 in oocyte maturation and illustrated some of the mechanisms by which Hfm1 takes part in the occurrence and development of POI. In conclusion, these results suggest that Hfm1 participates in Golgi-associated spindle assembly and division in mouse oocyte meiosis. We hope such findings provided better understanding of the relationship between HFM1 and POI that ultimately aid the advances in the treatments.

## Materials and methods

### Mouse breeding and fertility test of female mice

Mice carrying the a floxed (loxP-flanked) allele of Hfm1(hereafter referred to as Hfm1^fl/fl^) or the Gdf9-Cre transgenic (hereafter referred to as Gdf9-Cre) were produced from GemPharmatech Co;Ltd (Nanjing, China). These mice were maintained on identical C57BL/6J genetic background. Hfm1^fl/fl^;Gdf9-Cre mice were produced by crossing Hfm1^fl/fl^ mice with Gdf9-Cre transgenic mice (hereafter referred to as Hfm1-cKO) and Hfm1^fl/fl^ mice were used as control group. All animal experiments were approved by the institutional ethics committee of Nanjing Medical University and were performed by the National Institutes of Health Guide for the Care and Use of Laboratory Animals. Mice were genotyped by PCR using primers as shown in Supplementary Table 1 and Supplementary Fig. 1. Fertility tests were carried out by mating 2-month-old Hfm1-cKO and control females with normal adult C57BL/6J males until 9 months old. The number of pups for each litter was recorded at birth, and the average number of pups per litter was calculated at the end of the testing period. For each genotype, four females were used.

### Histology and immunofluorescence

For histology staining, ovaries from 2-month-old and 9-month-old mice were fixed in 4% paraformaldehyde at room temperature overnight, and stored at 4 °C in fresh 75% ethanol until further use. Ovaries were then dehydrated, embedded in paraffin, and 5 μm serial sections were stained with hematoxylin and eosin (H&E) using standard protocol. Ovaries from five females per group were used (*n* = 5). The follicles containing oocytes with a visible nucleus were counted in every fifth section of the entire ovary and were scored as primordial, primary, secondary, or antral follicles based on their morphological appearances as described previously^39^. Briefly, primordial follicles were classified as an oocyte surrounded by one layer of flattened granulosa cells; primary follicles were classified as an oocyte surrounded by one layer of cuboidal granulosa cells; secondary follicles were classified as an oocyte surrounded by more than one layer of cuboidal granulosa cells with no visible antrum; and antral follicle were classified as an oocyte surrounded by multiple layers of cuboidal granulosa cells and containing one or more antral spaces.

For immunofluorescence analysis, paraffin-embedded sections were dewaxed, and heat-mediated antigen retrieval was performed by microwaving for 20 min in 10 mM sodium citrate (pH 6.0) (P0083, Beyotime). The sections were cooled for 15 min, washed in deionized water and then rinsed twice in PBS. The sections were incubated in 5% goat serum for 30 min and then incubated overnight at 4 °C with a primary Rabbit antibody against Hfm1 (PA5-83256, 1:100, ThermoFisher), followed by incubation with CoraLite488 conjugated Goat Anti-Rabbit IgG(H + L) (SA00013-2, 1:200, Proteintech) for 1 h at room temperature. DAPI (C1006, Beyotime) was used for DNA counterstaining. Two random fields per slide (two slides per animal, three animals per group, *n* = 12) were examined. The signals were acquired by performing the same immunostaining procedure and setting up the same parameters with a confocal microscope (Nikon, Japan).

### Oocyte isolation and immunofluorescence

To obtain GV stage oocytes, 3-week-old female mice were primed with 5 IU pregnant mare’s serum gonadotropin (PMSG, Ningbo A Second Hormone Factory, China) 44–46 h prior to ovary collection. Ovaries were collected in M2 medium (M7167, Sigma) and supplemented with 2.5 μM milrinone (M4659, Sigma) to maintain meiotic arrest. Cumulus oocyte complexes were collected and oocytes were mechanically denuded of cumulus cells according to standard procedures.

To obtain MII oocytes, 3-week-old female mice were primed with PMSG (5 IU) and injected 5 IU human chorionic gonadotropin (hCG, Ningbo A Second Hormone Factory, China) 44 h later. After 14–16 h, MII oocytes were collected from the ampulla of oviducts.

For immunofluorescence, oocytes were fixed with 4% paraformaldehyde in phosphate-buffered saline (PBS, ThermoFisher) at room temperature for 30 min. They were then permeabilized with 0.3% Triton X-100 in PBS for 30 min and blocked with 0.3% BSA in PBS for 30 min. Oocytes incubated in a Rabbit anti-Hfm1 antibody (PA5-83256, 1:100, ThermoFisher), a Mouse anti-GM130 antibody (610822, 1:100, BD Biosciences), a Alexa Fluor 488 conjugate β-Tubulin (9F3) Rabbit antibody (3623, 1:200, CST) or a Rabbit anti-Phospho-p44/42 MAPK (Erk1/2) antibody (4370, 1:100, CST) overnight at 4 °C. The next morning, some oocytes were washed three times and incubated with CoraLite488 conjugated Goat Anti-Rabbit IgG(H + L) (SA00013-2, 1:200, Proteintech) or CoraLite594 conjugated Goat Anti-Mouse IgG(H + L) (SA00013-3, 1:200, Proteintech) for 1 h at room temperature. DAPI (C1006, Beyotime) was used for DNA counterstaining. TRITC-Phalloidin (MX4405,1:100, Cytoskeleton) was used for F-actin counterstaining. Oocytes were imaged using confocal microscope (Nikon, Japan). Every experiment was repeated three times and the data were analyzed using ImageJ software.

### In vitro maturation, fertilization, and embryo culture

For in vitro maturation, GV stage denuded oocytes were collected by stripping off the cumulus cells of cumulus–oocyte complexes as described previously. Denuded oocytes were cultured in M2 medium (M7167, Sigma), and samples of oocytes at GV, GVBD, Metaphase I (M I), and Metaphase II (MII) were then collected at the time points of 0, 4, 8, 14 h during culture.

For in vitro fertilization and embryo culture, MII oocytes were obtained from the ampulla of oviducts following standard hyperstimulation protocols and inseminated with normal sperm isolated from C57BL/6J adult males. Fertilized zygotes were confirmed by the presence of pronuclei, and they were then transferred into KSOM medium at 37 °C in a humidified atmosphere of 5% CO_2_ in air. The number of the zygotes, the 2-cell, 4-cell, 8-cell, and blastocyst stages were counted. For each genotype, four females were used.

### Western blot analysis

The proteins were extracted from mouse ovaries or oocytes from different stages using protein-loading buffer and heated at 100 °C for 5 min, then subjected to 10% SDS-PAGE. Separated proteins were transferred to a PVDF membrane. The membranes were blocked for 1 h at room temperature in TBST buffer [20 mM Tris, 137 mM NaCl, and 0.05% (w/v) Tween 20] containing 5% non-fat milk and were probed with specific rabbit polyclonal anti-Hfm1 (PA5-83256, 1:500, ThermoFisher), anti-Hfm1 (PA5-109810, 1:500, ThermoFisher), anti-Erk1/2 (11257-1-AP, 1:1000, Proteintech), anti- Phospho-p44/42 MAPK (Erk1/2) (4370, 1:1000, Cell Signaling Technology) and anti-Gapdh (10494-1-AP, 1:2000, Proteintech) antibodies at 4 °C overnight. After washing three times (10 min each) with TBST, the membranes were incubated at 37 °C with a HRP-conjugated Goat Anti-Rabbit IgG (H + L) (SA00001-2, 1:5000, Proteintech) for 1 h. After that, the membranes were washed three times in TBST and the blots were imaged using a ChemiDoc XRS + Molecular Imager (Bio-Rad) with Pierce ECL Western Blotting Substrate (32209, Thermo Fisher). To quantify Western blot results, band intensity values were measured by ImageJ software.

### Statistical analysis

All graphs and statistical analyses were generated using GraphPad Prism version 7 (GraphPad Software). All data are presented as the means ± SEM and were analyzed by Student’s *t*-test. Significance differences was defined as *P* < 0.05. A single asterisks (*) indicates a statistical difference at *P* < 0.05; double asterisks (**) indicate a statistical difference at *P* < 0.01; triple asterisks (***) indicate a statistical difference at *P* < 0.001.

## Supplementary information

Supplemental table 1

Supplementary figure 1

Supplementary figure 2

Supplementary figure and table legends
