# Supplementary material for: Hfm1 participates in Golgi-associated spindle assembly and division in mouse oocyte meiosis
Source: Cell Death Dis. 2020 Jun 30;11(6):490. doi: 10.1038/s41419-020-2697-4 (PMC7327073; doi:10.1038/s41419-020-2697-4)
Supplement: Supplementary file 1 — Supplemental table 1 [file 41419_2020_2697_MOESM1_ESM.docx]

**Supplementary Table 1**

**Primers used in genotype identification.**

| Annotation | Sequence | Results |
| --- | --- | --- |
| Loxp screening | F1:CACCTGATGTCTCCCAAGGATTCT | Fl: 339bp  WT: 248bp |
|  | R1:CACAGGTAATTCTGAGCTATGCTTG |  |
| Loxp locus | F2:TGTCTGGTCATGGCCAGGAATCTC | Fl: 1098bp  Wt: none |
|  | R2:CCAACTGACCTTGGGCAAGAACAT |  |
| Loxp and Target | F3:ATCCGGGGGTACCGCGTCGAG | Fl：2724bp  WT: none |
|  | R3:CACTCTCATGGCAATACTCCGATCCT |  |
| Gdf9-cre | F: TCTGATGAAGTCAGGAAGAACC | T: 500bp  WT: none |
|  | R: GAGATGTCCTTCACTCTGATTC |  |
