# Supplementary figures and images for: Hfm1 participates in Golgi-associated spindle assembly and division in mouse oocyte meiosis
Source: Cell Death Dis. 2020 Jun 30;11(6):490. doi: 10.1038/s41419-020-2697-4 (PMC7327073; doi:10.1038/s41419-020-2697-4)

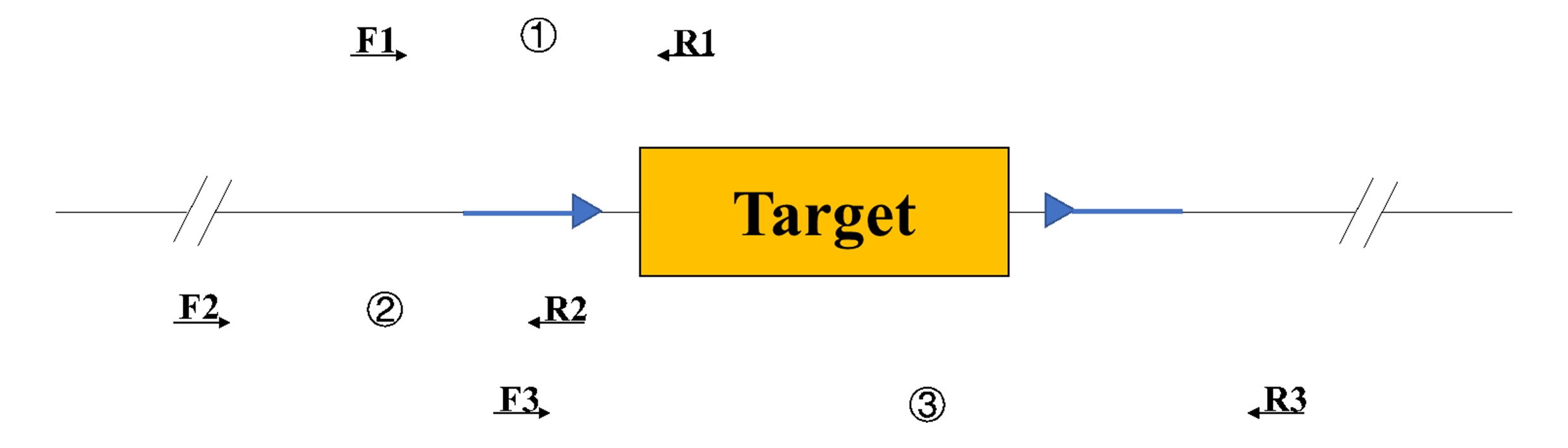

Supplement: Supplementary file 2 — Supplementary figure 1 [file 41419_2020_2697_MOESM2_ESM.tif]

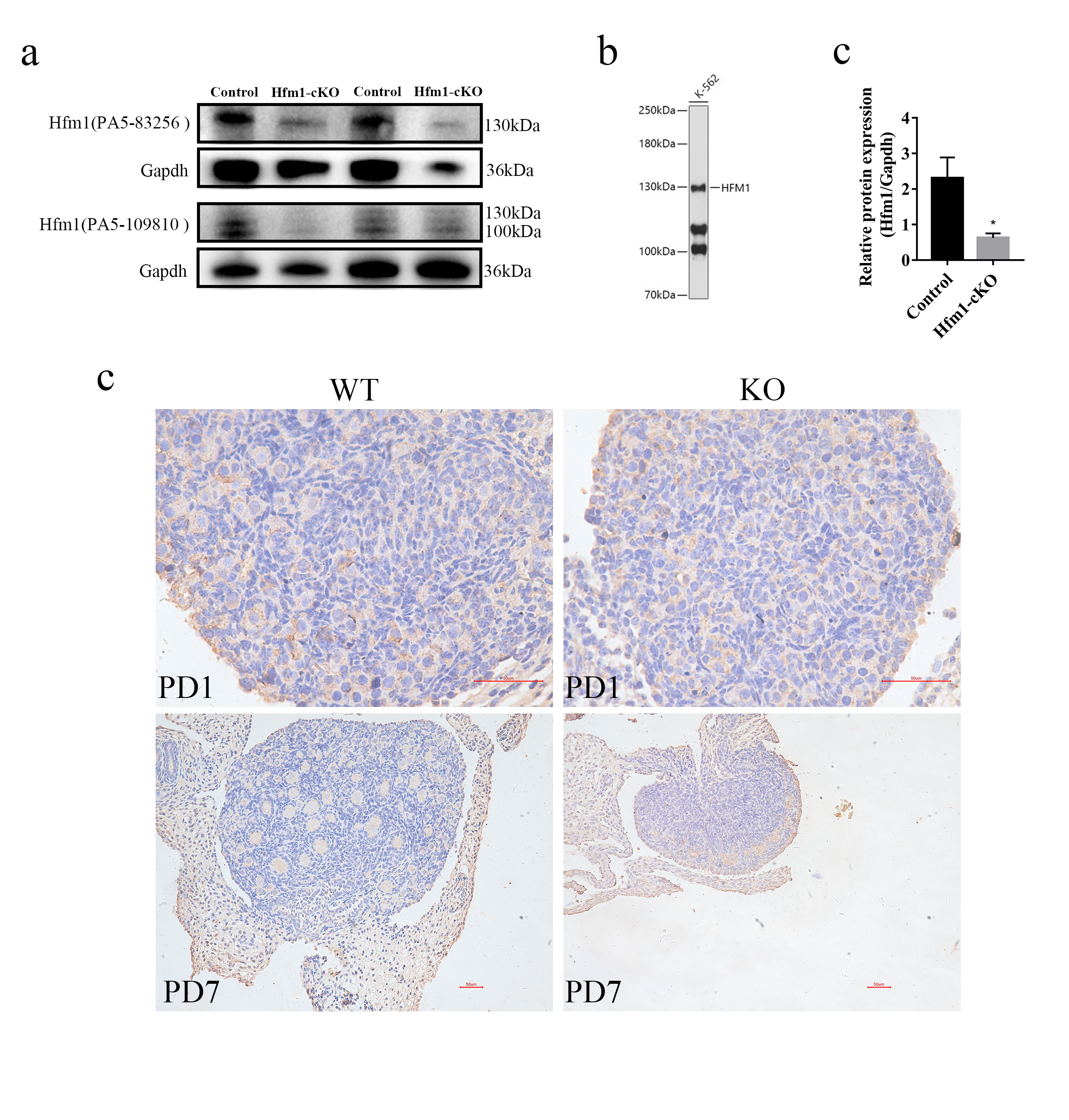

Supplement: Supplementary file 3 — Supplementary figure 2 [file 41419_2020_2697_MOESM3_ESM.tif]
