# Supplementary material for: Hfm1 participates in Golgi-associated spindle assembly and division in mouse oocyte meiosis
Source: Cell Death Dis. 2020 Jun 30;11(6):490. doi: 10.1038/s41419-020-2697-4 (PMC7327073; doi:10.1038/s41419-020-2697-4)
Supplement: Supplementary file 4 — Supplementary figure and table legends [file 41419_2020_2697_MOESM4_ESM.docx]

**Supplementary Figure legends**

**Supplementary table 1**

**Primers used in genotype identification.**

**Supplementary figure 1**

**Fig. S1. Schematic illustration of primers used in genotype identification.**

**Supplementary figure 2**

**Fig. S2. Related to Response letter.** **(a)** Western blot of Hfm1 expression in control and Hfm1-cKO oocytes at GV stage using two different Hfm1 polyclonal antibodies (PA5-83256, PA5-109810, ThermoFisher). Protein lysates from 100 oocytes were loaded in each lane. **(b)** Western blot results in K-562 cell using Hfm1 polyclonal antibodies (PA5-109810, ThermoFisher) from example in manual. **(c)** Quantitative analysis of Hfm1 protein expression levels (n=4). *p < 0.05. Significance was determined by two-tailed Student’s t tests. Data represent the mean ± SEM. **(d)** Immunohistochemistry (IHC) staining of Lhx8 in ovaries taken at postnatal day 1 (PD1) and postnatal day 7 (PD7) from Hfm1 whole-body knockout mice (KO) and wild type mice (WT).
